# Impact of Aortoseptal Angle Abnormalities and Discrete Subaortic Stenosis on Left-Ventricular Outflow Tract Hemodynamics: Preliminary Computational Assessment

**DOI:** 10.3389/fbioe.2020.00114

**Published:** 2020-02-27

**Authors:** Jason A. Shar, Kathleen N. Brown, Sundeep G. Keswani, Jane Grande-Allen, Philippe Sucosky

**Affiliations:** ^1^Department of Mechanical and Materials Engineering, Wright State University, Dayton, OH, United States; ^2^Department of Bioengineering, Rice University, Houston, TX, United States; ^3^Division of Pediatric Surgery, Texas Children’s Hospital, Houston, TX, United States; ^4^Department of Surgery, Baylor College of Medicine, Houston, TX, United States

**Keywords:** discrete subaortic stenosis, aortoseptal angle, left ventricular outflow tract, hemodynamics, fibrosis, wall shear stress, computational fluid dynamics

## Abstract

Discrete subaortic stenosis (DSS) is an obstruction of the left ventricular outflow tract (LVOT) due to the formation of a fibromuscular membrane upstream of the aortic valve. DSS is a major risk factor for aortic regurgitation (AR), which often persists after surgical resection of the membrane. While the etiology of DSS and secondary AR is largely unknown, the frequent association between DSS and aortoseptal angle (AoSA) abnormalities has supported the emergence of a mechanobiological pathway by which hemodynamic stress alterations on the septal wall could trigger a biological cascade leading to fibrosis and membrane formation. The resulting LVOT flow disturbances could activate the valve endothelium and contribute to AR. In an effort to assess this hypothetical mechano-etiology, this study aimed at isolating computationally the effects of AoSA abnormalities on septal wall shear stress (WSS), and the impact of DSS on LVOT hemodynamics. Two-dimensional computational fluid dynamics models featuring a normal AoSA (N-LV), a steep AoSA (S-LV), and a steep AoSA with a DSS lesion (DSS-LV) were designed to compute the flow in patient-specific left ventricles (LVs). Boundary conditions consisted of transient velocity profiles at the mitral inlet and LVOT outlet, and patient-specific LV wall motion. The deformation of the DSS lesion was computed using a two-way fluid-structure interaction modeling strategy. Turbulence was accounted for via implementation of the *k*-*ω* turbulence model. While the N-LV and S-LV models generated similar LVOT flow characteristics, the DSS-LV model resulted in an asymmetric LVOT jet-like structure, subaortic stenotic conditions (up to 2.4-fold increase in peak velocity, 45% reduction in effective jet diameter vs. N-LV/S-LV), increased vorticity (2.8-fold increase) and turbulence (5- and 3-order-of-magnitude increase in turbulent kinetic energy and Reynolds shear stress, respectively). The steep AoSA subjected the septal wall to a 23% and 69% overload in temporal shear magnitude and gradient, respectively, without any substantial change in oscillatory shear index. This study reveals the existence of WSS overloads on septal wall regions prone to DSS lesion formation in steep LVOTs, and the development of highly turbulent, stenotic and asymmetric flow in DSS LVOTs, which support a possible mechano etiology for DSS and secondary AR.

## Introduction

Discrete subaortic stenosis (DSS) is an obstruction to systolic blood flow resulting from the formation of a fibromuscular ring of tissue in the left ventricular outflow tract (LVOT) (Aboulhosn and Child, 2006; Foker, 2013). DSS occurs in 6% of children with congenital heart defects, and accounts for 8–30% of total LVOT obstructions in the pediatric population (Leichter et al., 1989; Donald et al., 2017). It’s associated with a spectrum of secondary pathologies among which the most prevalent is aortic regurgitation (AR), a condition defined by a reflux of diastolic blood back into the LV (Akinseye et al., 2018). Although 30–80% of DSS patients present with AR, the relationship between the two conditions is not well-understood (Van Der Linde et al., 2013). AR in DSS patients has been suggested to result from abnormalities in leaflet dynamics and long-term valvular damage caused by stenotic blood flow conditions (Foker, 2013; Pickard et al., 2015).

The current management of DSS focuses on eliminating the stenosis via surgical resection of the membrane. However, lesion recurrence within 10 years is high (8–34%) (Van Der Linde et al., 2013), which, when coupled with progressive AR, leads many patients to undergo multiple surgical interventions (Pickard et al., 2015). The elucidation of the etiology of DSS and secondary valvulopathy could alleviate some of the challenges posed by the long-term management of this disease. Unfortunately, to date, this knowledge remains limited. Although early studies classified DSS as a congenital disorder, its rare incidence in newborns and its lack of inheritance patterns have weakened the support for a genetic etiology (Cape et al., 1997; Lampros and Cobanoglu, 1998; Darcin et al., 2003). Alternatively, the frequent occurrence of DSS in LVs with a steep aortoseptal angle (AoSA, angle between the long axis of the aorta and the septal wall) has provided support to a mechanobiological etiology by which LV hemodynamic abnormalities could interact with the endocardium to drive fibrosis and DSS membrane formation (Cape et al., 1997; Yap et al., 2008; Foker, 2013; Massé et al., 2018). Little is also known about the mechanisms by which valvular functionality may be altered by the obstruction. *In vivo* studies have reported an asymmetric systolic leaflet flutter likely caused by alterations in LVOT hemodynamics induced by the lesion (Krueger et al., 1979; Foker, 2013). AR has also been described as the result of a wear mechanism caused by the mechanical interactions between the valve leaflets and the DSS membrane when the distance between those two structures allows for their periodic contact (Feigl et al., 1984; Nguyen, 2000). Lastly, a mechanobiological etiology has also been proposed in which DSS-induced LVOT flow alterations may subject the valve leaflets to stress abnormalities activating a biological state leading ultimately to inflammation, remodeling and ultimate AR (O’Leary and Wilkie, 2009; Foker, 2013).

The recent emergence of a possible mechano-etiology of DSS membrane formation and secondary valvulopathy justifies the need for a thorough characterization of the flow and hemodynamic stress abnormalities in DSS-prone and obstructed LVOTs. Previous attempts at assessing LV blood flow patterns have been made *in vivo* and *in vitro*. Phase-contrast magnetic resonance imaging (PC-MRI), which captures simultaneously and non-invasively anatomic structures and flow velocity, has evidenced the existence of complex ventricular flow patterns, and the formation of an asymmetric vortex ring during diastole, which is essential to the maintenance of normal cardiac function (Kim et al., 1995; Kilner et al., 2000; Gharib et al., 2006; Schenkel et al., 2009; Al-Wakeel et al., 2015). However, this measurement technique is still hampered by its limited spatial resolution, which restricts its use to the semi-quantitative assessment of macroscale flow features. *In vitro* particle-image velocimetry (PIV), which reconstructs the local velocity field in a two-dimensional (2D) domain from the displacement of tracer particles, has also been used to quantify the impact of various prosthetic heart valves on LV hemodynamics (Pierrakos et al., 2004; Fortini et al., 2013; Khalafvand et al., 2018; Saaid et al., 2018, 2019). This technique provides adequate spatial and temporal resolutions for the accurate assessment of the velocity field, turbulence characteristics and spatial velocity gradients, but challenges arise from the needs to maintain optical access to the model, and to build an experimental setup generating *in vivo*-like ventricular deformation.

Computational fluid dynamics (CFD), which consists of implementing numerical methods to solve the Navier-Stokes equations, has also been used to characterize LV hemodynamics. The widely used geometry-prescribed CFD strategy solves the flow equations in a domain whose geometry is prescribed at each time step, thereby neglecting the momentum transfer from the fluid to the domain boundary (Schenkel et al., 2009). This method has been implemented with idealized LV geometries (Baccani et al., 2002; Domenichini et al., 2005; Zheng et al., 2012; Arefin and Morsi, 2014) and patient-specific geometries acquired from cine MRI (Saber et al., 2001; Long et al., 2008; Doenst et al., 2009; Schenkel et al., 2009; Dahl et al., 2012). Three approaches have been proposed toward the characterization of LV wall motion and resulting hemodynamics using this strategy. The first approach, which consists of imposing a new mesh at each time step, is hampered by a poor accuracy due to the numerical errors resulting from the interpolation of the flow field from one grid to the next (Khalafvand et al., 2012; Nguyen et al., 2013; Caballero et al., 2017). To relax this challenge, another approach consists of applying directly local displacements to the fluid domain boundary via node-to-node mapping or correlation algorithms (Su et al., 2014b; Bavo et al., 2016; Lai et al., 2016; Doost et al., 2017). Although the fluid grid is progressively smoothed between time steps to maintain connectivity to the previous mesh, the resolution of the domain boundary may become compromised as the displacements of the boundary nodes are constrained by user inputs (Moosavi et al., 2014; Imanparast et al., 2016). As a third option, one-way fluid–structure interaction (FSI) techniques enable smooth changes between intermediate boundary nodes and the preservation of boundary grid resolution via automatic mapping of the local displacement nodes on the domain boundary (Moosavi et al., 2014; Imanparast et al., 2016). This approach has been successfully implemented to elucidate the impact of mitral inlet spatial velocity distributions on LV flow in three-dimensional (3D) models (Imanparast et al., 2016).

While CFD has been implemented extensively to capture the fluid mechanics of the normal LV, the impact of DSS or LVOT anatomical defects, which are presumed to play a role in DSS pathogenesis, have not been documented. In order to address this knowledge gap, the objective of the present study was to quantify computationally the flow and wall shear stress (WSS) characteristics in patient-specific LV models featuring (1) a normal LVOT, (2) an unobstructed LVOT with steep AoSA, and (3) an obstructed LVOT with steep AoSA and DSS lesion. The results of this characterization may provide new insights into the possible implication of AoSA abnormalities in DSS pathogenesis, and the validity of the mechano-etiology of DSS secondary valvulopathy.

## Materials and Methods

### LV Geometry Reconstruction

**FIGURE 1:**
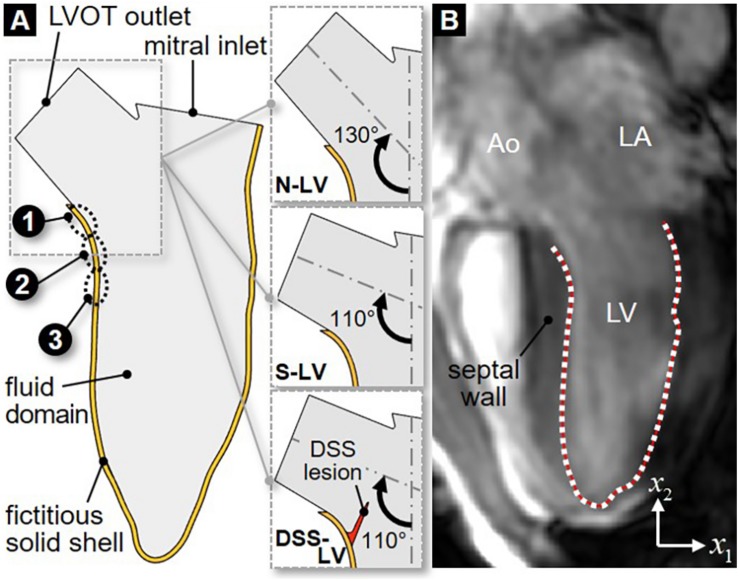
2D LV geometric models: **(A)** schematic describing the fluid domain and bounding fictitious solid shell geometries for the normal AoSA LV model (N-LV), the steep AoSA LV model (S-LV), and the steep AoSA + DSS lesion LV model (DSS-LV) (sites 1–3: WSS characterization sites); **(B)** LV wall segmentation from MRI image (Ao, aorta; LA, left atrium; LV, left ventricle; white dots, user-generated nodal points on the LV wall; red line, interpolating cubic spline).

The 2D LV model consisted of two domains: a solid domain representing the LVOT and LV walls, and a fluid domain representing the blood volume contained within the boundaries of the solid domain (Figure 1A). The LV anatomy was acquired from cardiac cine-MRI on a healthy 25-year old male volunteer using a 3T scanner (Discovery MR750w, GE Healthcare, Chicago, IL, United States). Images were captured from the 3-chamber view, with a field of view of 320 mm and a spatial resolution of 0.625 mm. 29 frames were acquired during each cardiac cycle of 0.772 s. Segmentation was performed using Segment v2.2 R6435 (Medviso AB, Lund, Sweden, Heiberg et al., 2010). The initial LV wall position was generated from the end-systolic contour using SolidWorks (Dassault Systèmes, Vélizy-Villacoublay, France). To improve the stability of the flow simulations, straight orifice extensions were added to the LVOT outlet (length: 10 mm; width: 18.6 mm) and mitral inlet (length: 2 mm; width: 24.4 mm). The papillary muscles and valvular structures were excluded. To assess the impact of the AoSA on LVOT WSS, two LV models referred to as N-LV and S-LV were constructed to reflect a normal (130°) and a steep (110°) AoSA, respectively (Figure 1A). Those angles were chosen based on echocardiography measurements in both control and DSS patients (Yap et al., 2008). To elucidate the impact of DSS on LVOT hemodynamics, a third geometry (DSS-LV) was constructed by attaching a compliant membrane (length: 10 mm; thickness: 1 mm) to the septal wall of the S-LV geometry, causing a 25% reduction in luminal diameter. This degree of stenosis is consistent with those described in two previous case reports, which indicated 26% and 40% reduction in LVOT luminal diameter (Ozsin et al., 2016; Kratochvíl et al., 2017). As the circular form of DSS typically involves the anterior mitral valve leaflet, a crescent-shaped obstruction was implemented to circumvent the exclusion of the valve.

### LV Wall Motion Acquisition

Manual segmentation of the LV chamber was performed by fitting a cubic spline to the portion of the LV wall located below the LVOT (Figure 1B). Repeating this process on each MRI image resulted in 29 spline curves reflecting the native deformation of the LV wall over one cardiac cycle. In order to allow for a hemodynamic assessment at a higher temporal resolution, the segmented LV contours were interpolated between two consecutive time frames (Schenkel et al., 2009). Briefly, each contour was discretized using 80 nodes and a total of 60 intermediate positions for each node between two consecutive contours were interpolated by fitting a cubic spline (Schenkel et al., 2009; Moosavi et al., 2014). This strategy generated 1,766 LV contours during one cardiac cycle and resulted in an effective temporal resolution of 0.4375 ms.

### Modeling Strategy

#### One-Way Fluid–Structure Interaction Framework

LV flow was computed by implementing a one-way FSI strategy using the commercial software ANSYS 19.2 (Canonsburg, PA, United States). Consistent with a methodology published previously (Imanparast et al., 2016), the fluid domain boundary was modeled in ANSYS Mechanical using a fictitious solid shell representation. In-plane displacement data (i.e., along the *x*_*1*_- and *x*_*2*_-directions, see coordinate system in Figure 1B) calculated between two consecutive frames were input as boundary conditions for each contour node at each time point to replicate the native deformation of the fluid domain boundary. The resulting displacement of the fictitious shell model was then transferred as a wall boundary condition in ANSYS Fluent using the ANSYS System Coupling module. The fluid domain was discretized using a dynamic mesh fitted to the moving LV wall and the flow governing equations were solved in their arbitrary Lagrangian Eulerian (ALE) form (Donea et al., 1982). At each time step, the fluid grid was updated to conform to the moving LV wall and the flow equations were solved iteratively until the criterion for numerical convergence was reached (continuity residual < 0.001). In order to capture the characteristics of the transitional LV flow, the flow equations consisted of the Reynolds-averaged continuity and Navier-Stokes equations:

$$\rho \,\frac{\partial \bar{u_{i}}}{\partial x_{i}}=0$$

and

$$\rho \,\frac{D\,\bar{u_{i}}}{D\,t}=-\frac{\partial \bar{p}}{\partial x_{i}}+\frac{\partial }{\partial x_{j}}\,\left(\mu \,\frac{\partial u_{i}}{\partial x_{j}}-\rho \,\bar{u_{i}^{\prime }\,u_{j}^{\prime }}\right),$$

respectively, where ρ is the fluid density, ${\bar{u}}_{i}$ is the ensemble-averaged velocity component along direction *x*_*i*_, $u_{i}^{\prime }$ is the velocity fluctuation, $\bar{p}$ is the ensemble-averaged pressure, and μ is the fluid dynamic viscosity. The components of the Reynolds stress $\rho \,\bar{u_{i}^{\prime }\,u_{j}^{\prime }}$ were calculated by implementing the shear stress transport (SST) *k*−ω turbulence model, which has been shown to effectively capture important features of LV flow such as adverse pressure gradients and flow separation (Menter, 1994; Kilner et al., 2000; Tabe et al., 2016). This model added two transport equations for turbulent kinetic energy, *k*, and specific dissipation rate ω:

$$\frac{\partial }{\partial t}\,\left(\rho \,k\right)+\frac{\partial }{\partial x_{i}}\,\left(\rho \,k\,{\bar{u}}_{i}\right)=\frac{\partial }{\partial x_{j}}\,\left(\Gamma_{k}\,\frac{\partial k}{\partial x_{j}}\right)+G_{k}-Y_{k}+S_{k}$$

and

$$\frac{\partial }{\partial t}\,\left(\rho \,\omega \right)+\frac{\partial }{\partial x_{i}}\,\left(\rho \,\omega \,{\bar{u}}_{i}\right)=\frac{\partial }{\partial x_{j}}\,\left(\Gamma_{\omega }\,\frac{\partial \omega }{\partial x_{j}}\right)+G_{\omega }-Y_{\omega }+D_{\omega }+S_{\omega },$$

respectively, where *G*_*k*_ is the generation of *k* due to mean velocity gradients, *G*_ω_ is the generation of ω, Г_*k*_ and Г_ω_ represent the effective diffusivity of *k* and ω, respectively, *Y*_*k*_ and *Y*_ω_ represent the dissipation of *k* and ω, respectively, *S*_*k*_ and *S*_ω_ are user-defined source terms, and *D*_ω_ represents the cross-diffusion term (Menter, 1994).

### Constitutive Models, Computational Grid and Boundary Conditions

#### Solid Domain

The solid fictitious shell, which was only used as a tool to transmit displacement data to the fluid domain boundary, was modeled as an isotropic, linear elastic material [density: 1000 kg/m^3^, Young’s modulus: 10 GPa, Poisson’s ratio: 0.001 (Imanparast et al., 2016)] in all three LV models. The shell was discretized with 449 elements, and the 80 displacement nodes generated during segmentation were mapped to the exterior surface.

In the DSS-LV model, the deformation of the DSS lesion was computed via a two-way FSI modeling strategy, which accounted for the transfer of momentum between the compliant membrane and the surrounding blood flow. Due to the lack of any prior mechanical characterization of DSS membrane tissue, the lesion was approximated as a nearly incompressible, isotropic, linear elastic material with properties matching those considered previously to model aortic valve leaflet mechanics [density: 1100 kg/m^3^, Young’s modulus: 0.37 MPa, Poisson’s ratio: 0.49 (Chandra et al., 2012)]. A mesh sensitivity analysis was performed in which the total deformation and von Mises stresses predicted in the lesion under steady peak-systolic flow conditions were compared using an increasingly refined lesion mesh featuring between 347 and 702 elements. The target criteria for mesh independence consisted of a change in total deformation and von Mises stresses smaller than 1% and 5%, respectively, between two consecutive grid sizes. Those criteria were met as the number of elements was increased from 556 to 702, which resulted in a 0.2% and 3.1% change in total deformation and von Mises stress, respectively. Therefore, the discretization of the lesion with 556 elements was deemed acceptable to yield mesh-independent results.

#### Fluid Domain

The fluid domain was discretized using a combination of unstructured wedge and hexahedral cells. A mesh sensitivity analysis was conducted to determine a domain discretization yielding mesh-independent results. The peak-filling velocity profile was captured along the LV short axis using four grids featuring a cell size between 142 and 500 μm. The correlation coefficient (*R*^2^) between velocity profiles predicted by two consecutive cell sizes was calculated and mesh independence was considered attained when *R*^2^ > 0.95. This criterion was satisfied as the cell size was reduced from 175 to 142 μm (*R*^2^ = 0.98), suggesting 175 μm as a suitable cell size for those simulations. The fluid mesh was refined in the vicinity of the wall (first cell height: 20 μm) to improve the prediction of turbulence characteristics in the viscous sublayer. This meshing strategy resulted in grid sizes of 177,600, 177,510, and 175,359 cells for the N-LV, S-LV and DSS-LV models, respectively. Consistent with previous LV flow simulations (Su et al., 2014a; Doost et al., 2017), blood was approximated as an incompressible, Newtonian fluid (ρ = 1050 kg/m^3^; μ = 0.0035 kg/m.s). The boundary conditions consisted of transient and spatially uniform velocity profiles at the mitral inlet and aortic outlet of the fluid domain. Diastolic ventricular filling (0 < *t* < 0.480 s) was modeled by imposing zero flow velocity at the aortic outlet to approximate aortic valve leaflet coaptation, and a transient velocity profile at the mitral inlet, whose instantaneous magnitude was calculated as the time-rate of change in LV volume. Conversely, during systolic ejection (0.480 < *t* < 0.772 s), a zero velocity was imposed at the mitral inlet, while a transient velocity profile calculated as the time-rate of change in LV volume was prescribed at the aortic outlet.

### Hemodynamic Endpoints

LV hemodynamics was characterized in terms of the mean in-plane velocity vector field (${\bar{\text{V}}}_{12}$) and the mean out-of-plane vorticity (${\bar{\Omega }}_{12}$) field defined as:

$${\bar{\text{V}}}_{12}={\bar{u}}_{1}\,{\hat{\text{e}}}_{1}+{\bar{u}}_{2}\,{\hat{\text{e}}}_{2}$$

and

$${\bar{\Omega }}_{12}=\left(\frac{\partial {\bar{u}}_{2}}{\partial x_{1}}-\frac{\partial {\bar{u}}_{1}}{\partial x_{2}}\right),$$

respectively. Turbulence characteristics were quantified in terms of turbulence kinetic energy (TKE) and Reynolds shear stress (RSS) defined as:

$$\text{TKE}=\frac{1}{2}\,\left({\bar{u}}_{1}^{2}+{\bar{u}}_{2}^{2}\right)$$

and

$$\text{RSS}=-\rho \,\bar{u_{1}^{\prime }\,u_{2}^{\prime }},$$

respectively.

To investigate the possible impact of AoSA abnormalities on the WSS environment in DSS-prone LV anatomies, WSS characteristics were captured in the N-LV and S-LV models over a region on the septal wall susceptible to DSS lesion formation (see Figure 1A). This region was discretized into three equally spaced 2-mm sub-regions: the crest of the septum (site 2), and the sites immediately above and below this location (site 1 and 3, respectively). WSS characteristics consisted of the instantaneous ensemble-averaged WSS component ${\bar{\tau }}_{12}$, the temporal shear magnitude (TSM), the temporal shear gradient (TSG) and the oscillatory shear index (OSI) defined as:

$${\bar{\tau }}_{12}=\mu \,\left(\frac{\partial {\bar{u}}_{1}}{\partial x_{2}}+\frac{\partial {\bar{u}}_{2}}{\partial x_{1}}\right),$$

$$\text{TSM}=\frac{1}{T}\,\int_{0}^{T}\left|{\bar{\tau }}_{12}\right|\,dt,$$

$$\text{TSG}=\frac{1}{T}\,\int_{0}^{T}\left|\frac{\partial {\bar{\tau }}_{12}}{\partial t}\right|\,dt,$$

and

$$\text{OSI}=\frac{1}{2}\,\left[1-\frac{\left|\int_{0}^{T}{\bar{\tau }}_{12}\,dt\right|}{\int_{0}^{T}\left|{\bar{\tau }}_{12}\right|\,dt}\right],$$

respectively, where *T* is the cardiac period. The TSM and TSG characterize the time-averaged magnitude and temporal gradients of the WSS over one cardiac cycle, respectively, while the OSI quantifies the oscillatory nature of the WSS signal (OSI = 0: purely pulsatile/unidirectional; OSI = 0.5: purely oscillatory/bidirectional).

## Results

All three models were run for four cardiac cycles to achieve temporal convergence. The data presented in this section was captured during the fourth cycle.

### Global Velocity and Vorticity Characteristics

**FIGURE 2:**
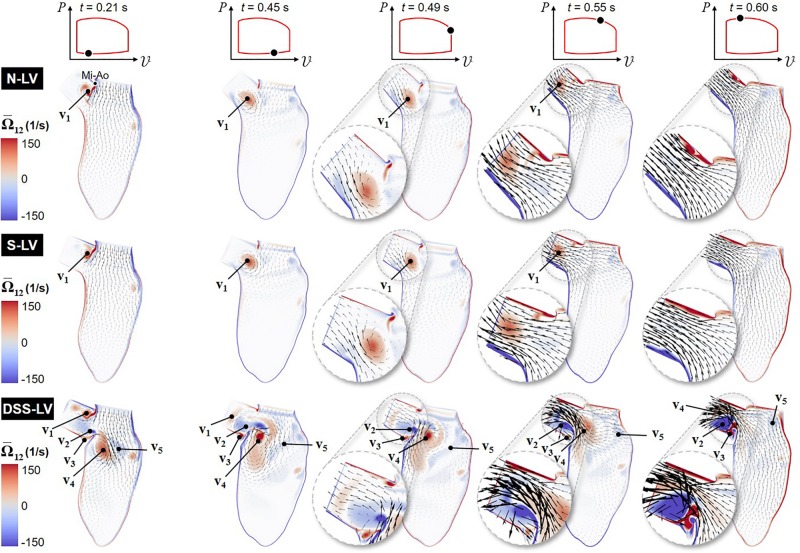
Snapshots of the velocity vector and vorticity contour fields captured in the N-LV, S-LV, and DSS-LV models during early ventricular filling (0.21 s), late filling (0.45 s), early systole (0.49 s), acceleration phase (0.55 s), and at peak systole (0.60 s) (Mi-Ao, mitral inlet-aortic outlet junction).

Animations of the mean velocity vector and vorticity contour fields predicted in all three models are included in Supplementary Video 1. Snapshots taken during early ventricular filling (E-wave), late filling (A-wave), early systole, acceleration phase, and at peak systole are shown in Figure 2.

The results suggest no substantial difference in flow patterns predicted by the N-LV and S-LV models. During early filling (*t* = 0.21 s), the increased convective acceleration of the flow emanating from the mitral inlet causes the flow to separate from the sharp junction between the mitral inlet and aortic outlet (Mi-Ao junction). The interaction between this shearing flow and the nearly stagnant LVOT flow results in the formation of a clockwise (CW) vortex within the LVOT (v_1_), whose size, position, and magnitude are qualitatively similar in both models. The deceleration of flow at the mitral inlet toward the end of ventricular filling (*t* = 0.45 s) is accompanied by the detachment of this vortex from the Mi-Ao junction and its migration toward the LVOT. During early ventricular contraction (*t* = 0.49 s), the CW vortex results in an asymmetric LVOT velocity distribution, characterized by high magnitudes near the septal wall and a velocity deficit on the aortic root side (see Figure 2 insets). The acceleration phase (*t* = 0.55 s) is associated with an attenuation in LVOT flow asymmetry due to the downstream migration of the vortex and its progressive destruction. At peak systole (*t* = 0.60 s), LVOT flows predicted in the N-LV and S-LV models exhibit the typical characteristics of flows through a contraction marked by an initial reduction in jet diameter up to the vena contracta followed by a downstream flow expansion and reattachment to the wall. The similarities in flow patterns between the two models are also supported quantitatively by the small (2.5%) difference in time-averaged vorticity predicted in the LVOT by both models.

The large deformations of the DSS membrane (DSS-LV model) generate substantial flow alterations dominated by the development of a rich and complex vortex dynamics. Throughout the cycle, the deflection of the DSS membrane and its interaction with LV flow give rise to alternating vortex shedding patterns from the tip of the lesion, whose frequency (∼25 Hz) is dramatically higher than that of the cardiac cycle (1.3 Hz). During early ventricular filling (*t* = 0.21 s), the lesion contributes to the entrapment within the LVOT of the same CW vortex as that observed in the N-LV and S-LV models (v_1_). The resulting increased flow rotationality drives the formation of a large but relatively weak counterclockwise (CCW) vortex in the lower part of the LVOT (v_2_), as well as a smaller CW vortex on the upper side of the DSS lesion (v_3_). The separation of the mitral jet from the tip of the lesion gives rise to two large interventricular vortices (v_4_ and v_5_) rotating CW and CCW, respectively. The A-wave (*t* = 0.45 s) is accompanied by the attenuation of vortex previously formed at the Mi-Ao junction (v_1_), its migration further downstream toward the LVOT outlet, and the growth of the CCW vortex previously formed in the lower part of the LVOT (v_2_). These changes result in the displacement of the CW interventricular vortex (v_4_) toward the septal wall on the lower side of the DSS lesion, and the breakdown of the CCW interventricular vortex (v_5_) into smaller structures dispersed throughout the LV. At the beginning of the ejection phase (*t* = 0.49 s), the reversal of the interventricular flow direction causes the destruction of the LVOT vortex (v_1_), flow separation at the tip of the lesion, and the strengthening of the CCW LVOT vortex trapped on the upper side of the DSS lesion (v_2_). During ejection (*t* = 0.55 s), the production of vorticity caused by the increased velocity gradient along the septal wall feeds the CCW vortex trapped behind the lesion (v_2_). The increased LV flow momentum results in the migration of this vortex and the CW interventricular vortex (v_4_) further downstream in the LVOT. This complex vorticity dynamics combined with the obstruction caused by the DSS lesion skews the flow toward the upper half of the LVOT, and generates a large recirculation bubble immediately downstream of the lesion. The resulting LVOT flow asymmetry, which is marked by high velocity magnitude near the upper half of the LVOT and low retrograde flow near the bottom half, is opposite to that observed in the N-LV and S-LV models. Throughout the acceleration phase (*t* = 0.55 s) and until peak systole (*t* = 0.60 s), the DSS lesion generates stenotic flow conditions in the LVOT, as suggested by the substantial increase in maximum flow velocity magnitude (up to 2.2- and 2.4-fold increase during acceleration phase and at peak systole, respectively) and reduction in effective LVOT jet diameter (45% reduction at peak systole) relative to the N-LV and S-LV models. The disturbed hemodynamics generated by the DSS lesion also resulted in a 2.8-fold increase in time-average vorticity in the LVOT relative to the normal LV (N-LV).

### LVOT Velocity Profile Characteristics

**FIGURE 3:**
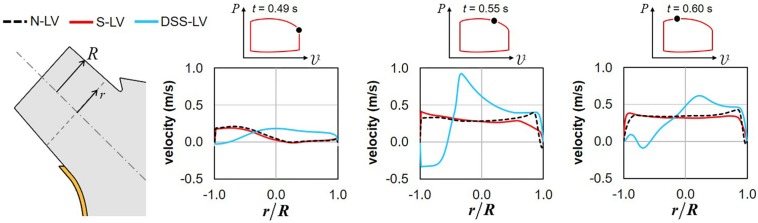
Snapshots of the velocity profiles captured in the N-LV, S-LV, and DSS-LV models at the base of the LVOT during early systole (0.49 s), acceleration phase (0.55 s), and at peak systole (0.60 s) (*r*: radial position; *R*: LVOT radius).

The time histories of the velocity profiles predicted at the base of the LVOT in the three models were compared during the systolic phase to isolate the respective impact of AoSA abnormalities and DSS on LVOT hemodynamics (Figure 3).

Confirming the trend observed on the velocity vector field, the N-LV and S-LV models exhibit qualitatively similar velocity profiles throughout the ejection phase. At the beginning of LV contraction (*t* = 0.49 s), the LVOT velocity profile is asymmetric with velocity overload close to the septal wall and velocity deficit on the opposite side. As the flow accelerates (*t* = 0.55 s) and until peak systole (*t* = 0.60 s), the degree of asymmetry becomes less pronounced, resulting in a nearly uniform velocity profile in the bulk of the flow and large velocity gradients constrained within the boundary layer region. Those characteristics are typical of the velocity profile for turbulent flow in pipes.

The presence of the DSS lesion resulted in substantial alterations of the velocity distribution. Although the DSS-LV model still predicts an asymmetric velocity distribution in early systole (*t* = 0.49 s), the velocity profile exhibits an inverted distribution as compared to the N-LV and S-LV predictions, with low-magnitude flow reversal near the septal wall and high-magnitude forward flow on the opposite side. Those spatial characteristics are associated with a reduction in maximum velocity magnitude (19% and 15% decrease vs. N-LV and S-LV, respectively). In contrast, later during the acceleration phase (*t* = 0.55 s) and at peak systole (*t* = 0.60 s), the DSS-LV model exhibits substantial increases in velocity magnitude as compared to the unobstructed models (up to 2.4- and 2.2-fold increase during the acceleration phase and at peak systole, respectively).

### Turbulence Characteristics

#### Turbulence Kinetic Energy

**FIGURE 4:**
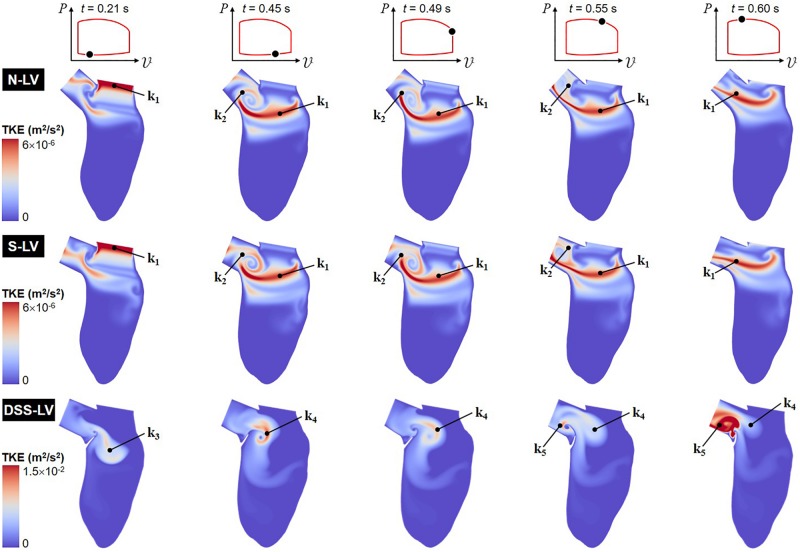
Snapshots of the TKE contour fields captured in the N-LV, S-LV and DSS-LV models during early ventricular filling (0.21 s), late filling (0.45 s), early systole (0.49 s), acceleration phase (0.55 s), and at peak systole (0.60 s).

Animation showing the spatial distributions of TKE throughout one cardiac cycle is included in Supplementary Video 2. Snapshots captured at five phases of the cardiac cycle are shown in Figure 4.

The TKE distribution exhibit some of the characteristics of the mean velocity distribution. Regardless of the model, the TKE is small near the LV wall and the DSS lesion due to the suppression of turbulence near solid boundaries and the low magnitude of velocity fluctuations in those regions. The N-LV and S-LV models exhibit similar TKE distributions with only moderate indication of flow turbulence (max TKE = 6 × 10^–6^ m^2^/s^2^). Highest TKE levels are observed at the beginning of ventricular filling (*t* = 0.21 s) immediately downstream of the mitral valve orifice as a result of the velocity fluctuations caused by the valve opening (region k_1_). At the end of the filling phase (*t* = 0.45 s) and during early ejection (*t* = 0.49 s), the region of highest TKE migrates downstream within the LV, while stretching horizontally. Consistent with the vorticity predictions during the same period, another region characterized by similar TKE levels (region k_2_) is also present in the shear layer bounding the CW vortex formed within the LVOT (v_1_). Those two regions result in a large horizontal band of elevated TKE extending from the posterior side of the LV to the septal wall connected to a smaller circular region rolling within the LVOT. Throughout the acceleration phase (*t* = 0.55 s) and until peak systole (*t* = 0.60 s), the opening of the aortic valve and the increased momentum of the LV flow wash away the LVOT vortex, causing the migration of the large velocity fluctuations concentrated in its bounding shear layer (region k_2_) toward the LVOT outlet, while high TKE levels persist along the LVOT axis (region k_1_).

The presence of the DSS membrane results in a dramatic increase in flow turbulence, as suggested by the five-order-of-magnitude increase in peak TKE predicted in the DSS-LV model (max TKE = 1.5 × 10^–2^ m^2^/s^2^) relative to the N-LV and S-LV models. In addition, the large deflection of the membrane throughout the cardiac cycle results in complex TKE distributions whose characteristics closely mimic those of the vorticity dynamics captured in the DSS-LV model. At the beginning of ventricular filling (*t* = 0.21 s), moderate TKE levels are present over a large region (region k_3_) consisting of the region sandwiched between the two interventricular vortices (v_4_ and v_5_), and the shear layer bounding the bottom of those vortical structures. The end of the filling phase (*t* = 0.45 s) and early ejection (*t* = 0.49 s) are accompanied by the destruction of TKE in this region and a production of TKE in the region separating the two vortices (v_2_ and v_4_) formed on both sides of the lesion (region k_4_). Throughout the acceleration phase (*t* = 0.55 s) and until peak systole (*t* = 0.60 s), the deflection of the DSS membrane toward the LVOT and the migration of the two prominent vortices (v_2_ and v_4_) toward the LVOT result in turbulence weakening in region k_4_ and production of high-intensity turbulence in the LVOT (region k_5_).

#### Reynolds Shear Stress

**FIGURE 5:**
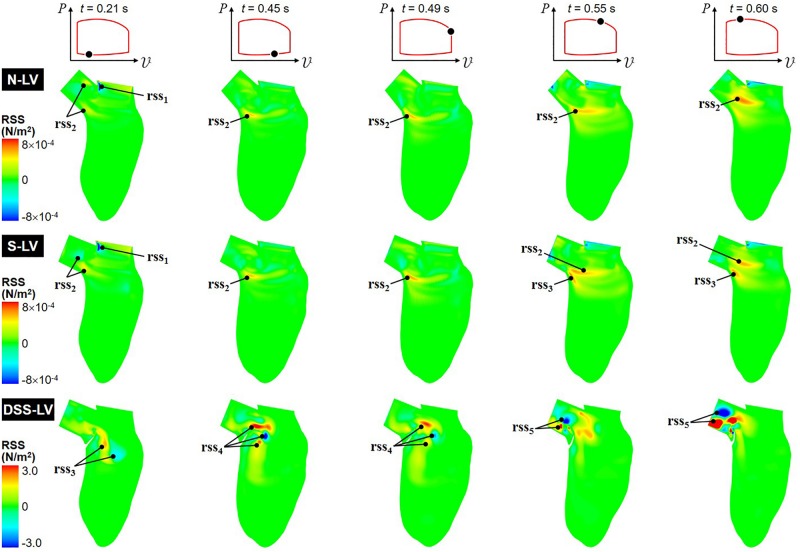
Snapshots of the RSS contour fields captured in the N-LV, S-LV, and DSS-LV models during early ventricular filling (0.21 s), late filling (0.45 s), early systole (0.49 s), acceleration phase (0.55 s), and at peak systole (0.60 s).

Animation of the RSS field, which characterizes the impact of the turbulence on the mean flow (Tennekes and Lumley, 1972), is included in Supplementary Video 3, while snapshots captured at five phases of the cardiac cycle are shown in Figure 5.

The N-LV and S-LV models exhibit similar RSS distributions, with lowest RSS levels near the apical LV region and larger values (up to 8 × 10^–4^ N/m^2^) concentrated in the vicinity of and within the LVOT. During early ventricular filling (*t* = 0.21 s), the RSS is largest in the shear layer extending from the sharp Mi-Ao junction (rss_1_). More moderate RSS levels can also be observed in the shear layer bounding the vortex trapped within the LVOT (rss_2_). As expected, the RSS remains low near the LV wall where turbulence is inhibited. From the end of ventricular filling until the acceleration phase (0.45 s < *t* < 0.55 s), the deceleration of the mitral jet causes the dissipation of the high RSS levels previously observed near the Mi-Ao junction, while the increased circulation of the CW vortex (v_1_) is accompanied by a progressive increase in RSS in the bottom of the shear layer bounding this vortical structure (rss_2_). Interestingly, during acceleration phase (*t* = 0.55 s), the steeper AoSA results in the formation of a second high-RSS region near the crest of the septal wall (rss_3_), which results from the interaction between the upward LV blood flow and the rotational flow trapped at the base of the LVOT. At peak systole (*t* = 0.60 s), the high-momentum ejection and redirection of the blood from the LV to the LVOT results in the growth and intensification (up to 6 × 10^–4^ N/m^2^) of the high-RSS region (rss_2_), and its migration toward the axis of the LVOT.

The DSS-LV model exhibits dramatic alterations in RSS characteristics, which correlate to the complex vortex shedding associated with the periodic deflection of the lesion. The peak RSS level predicted in this model (3.0 N/m^2^) represents an increase of more than three orders of magnitude relative to the levels computed in the absence of DSS lesion. During early filling (*t* = 0.21 s), high RSS levels are concentrated in the vicinity of the two interventricular vortices described previously (rss_3_). In late filling (*t* = 0.45 s), the interaction between the mitral jet and the deflection of the DSS membrane toward the LV chamber generate high RSS levels (3.0 N/m^2^) concentrated in multiple pockets located at a distance from the lesion near the base of the LVOT (rss_4_). While those pockets persist until early ejection (*t* = 0.49 s), they are characterized by a substantial (up to 47%) reduction in RSS levels. The deflection of the DSS membrane toward the LVOT and its fluttering during the systolic phase (0.55 s < *t* < 0.60 s) form two main regions of high RSS (rss_5_), which relate to the two vortical structures (v_2_ and v_4_) observed in the LVOT during this phase.

### Septal WSS Characteristics

**FIGURE 6:**
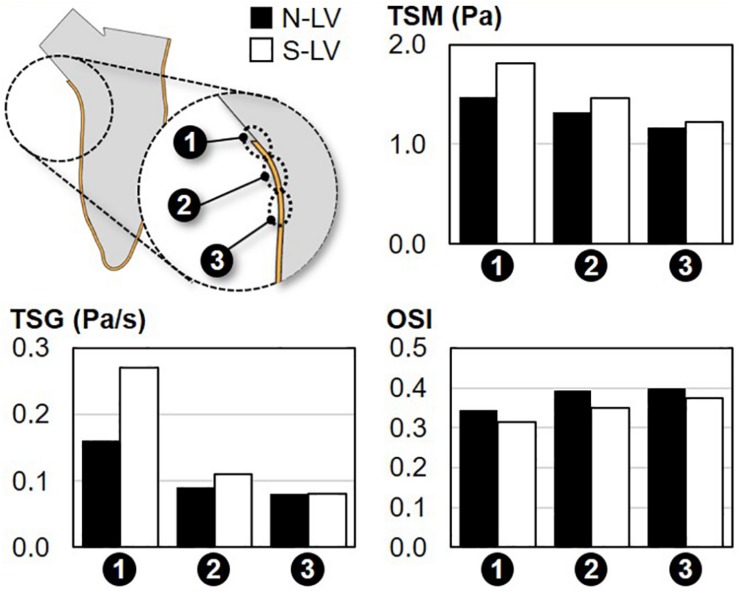
Comparison of the TSM, TSG and OSI on the septal wall of the N-LV and S-LV models (site 1: immediately above the septal wall crest; site 2: septal wall crest; site 3: immediately below the septal wall crest).

The WSS characteristics captured along the septal wall in the N-LV and S-LV model are shown in Figure 6.

#### Temporal Shear Magnitude

Regardless of the AoSA, the TSM predictions suggest a net increase in WSS magnitude along the septal wall when approaching the LVOT (26% and 48% increase in TSM between site 3 and site 1 in N-LV and S-LV, respectively). The maximum TSM, which occurs at site 1 (i.e., region closest to the LVOT) in both models, is three-order-of-magnitude higher than the predicted peak RSS value. This observation is in agreement with the typical structure of turbulent flows in the viscous sublayer where laminar shear stress dominates over turbulent shear stress. Importantly, the steep AoSA model consistently subjects the septal wall to a WSS overload as compared to the normal AoSA model (23%, 10%, and 5% increase in TSM in S-LV vs. N-LV at site 1, site 2, and site 3, respectively).

#### Temporal Shear Gradient

Consistent with the TSM predictions, WSS conditions are increasingly harsh in the proximity of the LVOT, as illustrated by the 100% and 238% increase in TSG between site 3 and site 1 in the N-LV and S-LV model, respectively. Also consistent with the TSM observations is the increasingly adverse impact of a steep AoSA on the TSG experienced by the endocardium from site 3 to site 1 (0%, 22%, and 69% difference in TSG between N-LV and S-LV at site 3, site 2 and site 1, respectively).

#### Oscillatory Shear Index

The analysis of the OSI reveals the weak spatial- and AoSA-dependence of the WSS directionality along the septal wall. Both the N-LV and S-LV models predict the existence of a WSS environment with some degree of bidirectionality at all three sites (OSI > 0.31). The convergence of the blood flow toward the LVOT during systole contributes to a slight increase in WSS unidirectionality in the proximity of the LVOT (-0.06 point in OSI between site 3 and site 1 in N-LV and S-LV).

## Discussion

In this computational analysis, blood flow patterns were predicted in realistic deforming LV models to elucidate the impact of AoSA abnormalities and DSS on LVOT hemodynamics. The main contributions of the study are: (1) the demonstration of causality between AoSA abnormalities and WSS overloads in septal wall regions prone to DSS lesion formation; and (2) the development of stenotic, disturbed and rotational hemodynamics in DSS LVOTs.

### AoSA Steepening Subjects the Septal Wall to WSS Overloads

In contrast to its limited impact on overall LVOT flow structure, LVOT steepening resulted in a substantial alteration of the WSS environment on the septal wall in terms of both magnitude and temporal gradients. A decrease in AoSA from 130° to 110° was accompanied by up to 23% and 69% increase in TSM and TSG, respectively. Given the wide range of AoSA values reported in the literature (Kleinert and Geva, 1993; Yap et al., 2008; Barboza et al., 2013), more dramatic mechanical stress alterations might be expected in some cases. In contrast, the steepening of the AoSA did not alter the oscillatory characteristics of the WSS, as suggested by the relatively similar OSI values predicted by the N-LV and S-LV models. This sensitivity of the LVOT spatial velocity distribution on septal wall anatomy has already been reported clinically (Zhou et al., 1996). The present results are also in agreement with a previous computational study suggesting an increase in near-wall spatial velocity gradient with decreasing AoSA in a simplified LV geometry (Cape et al., 1997). Interestingly, the magnitude of the WSS overloads reported in the present study near the crest of the septal wall (up to 23% vs. normal AoSA) is also quantitatively similar to the values reported during systole (up to 24% vs. normal AoSA) in a simplified 3D LV model published by our group (Massé et al., 2018).

### DSS Alters Interventricular Hemodynamics

Despite the limitations of the 2D approach adopted in the present study, the unobstructed LV models (N-LV and S-LV) were able to capture the diastolic asymmetric interventricular vortex previously reported *in vivo* and in computational studies (Kilner et al., 2000; Schenkel et al., 2009; Le and Sotiropoulos, 2012; Su et al., 2014a; Bavo et al., 2016). This vortex, which results from the expansion of the mitral jet into the LV chamber and its separation from the anterior leaflet (Khalafvand et al., 2012; Su et al., 2014b), enhances ventricular ejection by redirecting fluid away from the LVOT during ventricular filling (Kilner et al., 2000). Consistent with previous observations (Cape et al., 1997; Dahl, 2012; Khalafvand et al., 2012), the velocity profiles captured in the unobstructed LVOTs exhibited relatively uniform distributions with strong spatial gradients in the viscous sublayer, which are typical of turbulent flows in pipes. The obstructed LVOT exhibited a very contrasted interventricular hemodynamics marked by a larger diastolic interventricular vortex, its migration further toward the apex of the LV chamber, and the formation of several smaller vortices throughout the filling phase. Interestingly, those flow features are qualitatively similar to those reported in a previous study examining outflow obstruction caused by hypertrophic cardiomyopathy, which evidenced the formation of a dominant anterior vortex ring and its migration toward the center of the LV chamber, and the formation of several smaller vortices in the LVOT (Su et al., 2014a).

### DSS Generates Stenotic Flow and Disturbed Vorticity Dynamics in the LVOT

An important characteristic captured by the DSS-LV model was the dramatic increase in flow turbulence in the LVOT relative to the unobstructed models. While this observation is consistent with previous clinical studies, which have described turbulence during systolic ejection as a hallmark of DSS (Aboulhosn and Child, 2006; Foker, 2013; Van Der Linde et al., 2013), the present study provides new insights into the mechanisms of turbulence production. First, the DSS lesion resulted in stenotic conditions upstream of the aortic valve, marked by a net increase in LVOT flow velocity and skewness, which contribute to flow instability and the emergence of fluctuating velocity components. Those characteristics, which result from the redirection of the flow around the lesion and the formation of a jet-like structure in the LVOT, have been documented in color Doppler echocardiography studies (Aboulhosn and Child, 2006; Opotowsky et al., 2017). Importantly, the present study reveals that those flow characteristics result in an effective degree of stenosis in DSS that is larger than that due to the physical occlusion imposed by the lesion (45% reduction in effective luminal diameter at the vena contracta vs. 25% at the tip of the lesion). This result has an important clinical significance as it suggests that: (1) a distinction should be made between the geometric orifice area at the tip of the lesion (GOA) and the effective orifice area at the waist of the LVOT jet (EOA); and (2) the grading of stenosis in DSS patients should be based on velocity criteria rather than lesion dimensional criteria by analogy with the assessment of valvular stenosis, which accounts for flow energy loss across the valve (Seaman and Sucosky, 2014). A second mechanism of turbulence production may stem from the complex vorticity dynamics observed in the LVOT, especially during ejection. The interactions between the DSS membrane deflection and blood flow redirection between ventricular filling and systolic ejection gave rise to high-frequency alternating vortex shedding from the tip of the lesion, which amplifies flow instability. It is important to note that the flow characteristics captured by the DSS-LV model were not observed in the absence of lesion even with a steep AoSA (i.e., S-LV model). This suggests that flow stenosis and turbulence, which have been previously described as the hallmarks of DSS hemodynamics, are solely the results of the DSS membrane at the base of the LVOT.

### Potential Significance for DSS Pathogenesis and Secondary AR

The WSS overloads captured near the crest of the septal wall in the steep LVOT provide new insights into the possible role played by mechanical stresses in DSS pathogenesis. Disturbed flow has been shown to alter endothelial cell-smooth muscle cell communication and behavior in blood vessels (Ando and Yamamoto, 2011; Shav et al., 2014; Chistiakov et al., 2016). For example, *ex vivo* experiments with porcine tissue have shown that WSS magnitude overloads on the aortic wall secondary to valvular defects contribute to the loss of tissue homeostasis and the progressive degeneration of the tunica media (Atkins and Sucosky, 2014; Atkins et al., 2014, 2016). In light of those observations, the increased WSS magnitude and temporal gradient captured on the septal wall may promote fibrosis and explain the frequent association between DSS and steep LVOTs documented in the literature (Cape et al., 1997; Yap et al., 2008; Foker, 2013; Massé et al., 2018). In addition, *in vitro* studies in a step-flow channel have evidenced increased cellular proliferation in flow reattachment regions subjected to high WSS spatial gradients (Tardy et al., 1997; Chien, 2003). Interestingly, the WSS analysis conducted in the present study at three sites on the septal wall indicates that WSS overloads (TSM and TSG) and high spatial gradients not only exist in the steep LVOT, they also seem to increase in the streamwise direction (TSM change from site 3 to site 2: 0.15 and 0.24 Pa in N-LV and S-LV, respectively; TSM change from site 2 to site 1: 0.15 and 0.35 Pa in N-LV and S-LV, respectively). While studies are needed to verify the sensitivity of endocardial cells to the WSS characteristics of the steep LVOT, the present results suggest the existence of a critical region on the septal wall where WSS alterations are maximized, which may also coincide with fibrosis and DSS lesion formation.

The present computational flow characterization also sheds new light into the possible role played by DSS hemodynamics on AR. The fluid mechanics generated during ejection by the DSS lesion was characterized by a jet skewed toward the upper wall of the LVOT. While the model did not include the aortic valve structure, this jet is likely to subject the leaflets to an asymmetric pressure distribution, which could result in asymmetric leaflet opening/closure dynamics and, ultimately, valvular insufficiency. While no study to date has examined leaflet-dependent damage/remodeling in DSS patients, the potential impact of DSS hemodynamics on aortic valve tissue has been suggested in a recent study, which reported the presence of an irregular fibrous thickening on a resected aortic valve (Lin, 2015). In addition to promoting asynchronous leaflet dynamics, the asymmetry of the LVOT flow caused by DSS may subject the valve leaflets to WSS abnormalities. Similarly to the vascular endothelium, the leaflet endothelium is particularly sensitive to its hemodynamic stress environment. WSS overloads on porcine aortic valve leaflets have been shown to mediate inflammation, remodeling and osteogenesis *ex vivo* (Sucosky et al., 2009; Hoehn et al., 2010; Balachandran et al., 2011; Sun et al., 2013; Sun and Sucosky, 2015). The stenotic flow conditions combined with the jet asymmetry captured in the DSS LVOT are expected to subject at least one leaflet to stress concentrations. Computational models incorporating a functional aortic valve are currently being developed to further assess the validity of this hypothetical pathway. The impact of stress abnormalities on leaflet inflammation and remodeling could be determined using the same bioreactors as those implemented to study the biological response of valvular tissue to time-varying WSS signals (Sucosky et al., 2008; Sun et al., 2011; Liu et al., 2019).

### Modeling Assumptions and Limitations

#### Dimensionality and Geometry

Although the 2D models implemented patient-specific geometries and LV wall deformation obtained from clinical images, LV hemodynamics would be better captured by 3D models. However, it is important to note that the 2D models were able to capture the typical flow features observed in the LV, and to evidence changes in septal WSS environment and LVOT hemodynamics in the presence of a DSS lesion or a steep AoSA, despite the prescription of similar LV deformations and valvular conditions. Besides, while the quantitative WSS results obtained in 2D may not accurately reflect the levels experienced in the native 3D environment, the qualitative features and trends captured by the simplified models are not expected to differ substantially from those observed in 3D. In fact, the 2D models effectively resolved the main flow components and the dominant WSS component in the plane of observation, as suggested by the close agreement between the predicted increase in LVOT velocity magnitude in the DSS-LV model (23% increase vs. normal AoSA) and that reported in our previous simplified 3D model (24% increase vs. normal AoSA; Massé et al., 2018). Nevertheless, 3D modeling would enable the prediction of the two WSS components on the septal wall and a more complete characterization of the LVOT vortex dynamics. The exclusion of the mitral and aortic valves was another simplification made in the models to reduce the complexity of the simulations. The absence of those structures prevented the examination of the impact of the membrane-aortic valve distance on valvular function and leaflet dynamics. Since this distance has been proposed as a major risk factor for progressive AR (Pickard et al., 2015), the future investigation of the effect of any anatomical parameter on valvular function will certainly benefit from the implementation of a 3D model incorporating a functional aortic valve.

#### Boundary Conditions and Blood Viscous Model

The prescription of the same LV deformation and inlet/outlet valve conditions in all three models was needed to effectively isolate the impact of LV anatomical abnormalities on LVOT hemodynamics. However, those conditions also resulted in imposing the same cardiac output in the three models. While this assumption may be acceptable in the N-LV and S-LV models, the flow disturbances caused by the DSS lesion are expected to generate a substantial energy loss, which could translate into a decreased cardiac function. However, this acute alteration in LV function is typically followed by the progressive recovery to normal hemodynamics as a result of LV remodeling. Therefore, the prescription of normal velocity conditions in the DSS case mimics the expected hemodynamics resulting from this long-term adaptation. A Newtonian fluid approximation was also used in the simulations. As demonstrated in another computational study, which compared left ventricular hemodynamics using a Newtonian formulation and various non-Newtonian models (Doost et al., 2015), peak-systolic LV flow characteristics are weakly impacted by non-Newtonian viscous effects, suggesting the suitability of the Newtonian viscous approximation in the present study.

#### DSS Lesion Morphological and Material Characteristics

It is important to note that the geometry of the DSS lesion modeled in this study does not reflect the wide spectrum of anatomies and DSS cases reported in the literature. As a result, the data collected may not be extrapolated to cases involving other disorders or subaortic stenosis morphotypes. In fact, the large variations in AoSA, DSS configuration and severity, and lesion location reported in the literature (Kleinert and Geva, 1993; Van Der Linde et al., 2013; Pickard et al., 2015) are likely to generate differential responses not captured by the current model. While a study could be conducted to investigate the full spectrum of subaortic stenosis phenotypes, or concomitant LV, aortic valve, or mitral valve disorders, the aim of the present study to isolate the impact of AoSA steepening on LVOT hemodynamics required to eliminate any other parameter that may have also altered the hemodynamic endpoints. By maintaining the same LV geometry in all cases, the results clearly demonstrate the ability of mild AoSA alterations to generate substantial vortex dynamics abnormalities in the LVOT and WSS alterations on the septal wall. In addition, the FSI strategy approximated the lesion as a linear elastic and isotropic material, with elastic properties similar to those used in prior valve studies. Although valve leaflets and DSS lesion tissue may not share the same mechanical properties, this assumption was needed to circumvent the lack of mechanical characterization of DSS membranes. Based on our observations from echocardiography images, the DSS lesion exhibits very large cyclic deformations and deflections, at a higher frequency than that of the cardiac cycle. This behavior was appropriately captured by the simplified material formulation.

## Data Availability Statement

The datasets generated for this study are available on request to the corresponding author.

## Author Contributions

PS, JS, KB, SK, and JG-A conceived the work. JS designed the models. JS and PS analyzed the data and wrote the manuscript. PS, KB, SK, and JG-A edited the manuscript.

## Conflict of Interest

The authors declare that the research was conducted in the absence of any commercial or financial relationships that could be construed as a potential conflict of interest.
