# Supplementary material for: Impact of Aortoseptal Angle Abnormalities and Discrete Subaortic Stenosis on Left-Ventricular Outflow Tract Hemodynamics: Preliminary Computational Assessment
Source: Front Bioeng Biotechnol. 2020 Feb 27;8:114. doi: 10.3389/fbioe.2020.00114 (PMC7056880; doi:10.3389/fbioe.2020.00114)
Supplement: Supplementary file 1 [file Data_Sheet_1.DOCX]

Supplementary Material

**Video1_velocity_vorticity.mp4** Animation of velocity vector and vorticity contour fields in the N-LV, S-LV and DSS-LV models over one cardiac cycle. Playback speed: 0.25×. Black moving ball indicates the current phase of the cardiac cycle on the pressure-volume diagram.

**Video2_TKE.mp4** Animation of TKE contour field in the N-LV, S-LV and DSS-LV models over one cardiac cycle. Playback speed: 0.25×. Black moving ball indicates the current phase of the cardiac cycle on the pressure-volume diagram.

**Video3_RSS.mp4** Animation of RSS contour field in the N-LV, S-LV and DSS-LV models over one cardiac cycle. Playback speed: 0.25×. Black moving ball indicates the current phase of the cardiac cycle on the pressure-volume diagram.
